# Supplementary material for: Machine learning-enabled exploration of the electrochemical stability of real-scale metallic nanoparticles
Source: Nat Commun. 2023 May 25;14:3004. doi: 10.1038/s41467-023-38758-1 (PMC10213026; doi:10.1038/s41467-023-38758-1)
Supplement: Supplementary file 1 — Supplementary Information [file 41467_2023_38758_MOESM1_ESM.docx]

Supplementary Information for

**Machine Learning-Enabled Exploration of the Electrochemical Stability of Real-Scale Metallic Nanoparticles**

Kihoon Bang,^1,2^ Doosun Hong,^1^ Youngtae Park,^1^ Donghun Kim,^2,^* Sang Soo Han,^2,^* and Hyuck Mo Lee^1,^*

^1^Department of Materials Science and Engineering, Korea Advanced Institute of Science and Technology (KAIST), Daejeon 34141, Republic of Korea

^2^Computational Science Research Center, Korea Institute of Science and Technology (KIST), Seoul 02792, Republic of Korea

*Correspondence to: Prof. Hyuck Mo Lee (hmlee@kaist.ac.kr), Dr. Sang Soo Han (sangsoo@kist.re.kr), Dr. Donghun Kim (donghun@kist.re.kr)

Keywords: *Surface Pourbaix diagram, Machine learning, Electrocatalysis, Surface stability*

**Supplementary Table S1. Elemental properties used for the atom feature vector** $\boldsymbol{v}_{\boldsymbol{i}}$ **in bond-type embedded CGCNN (BE-CGCNN) developments.**

| **Property** | **Range** | **Unit** | **Category #** |
| --- | --- | --- | --- |
| Group number | 1, 3 – 12, 15 |  | 12 |
| Period number | 1 – 6 |  | 6 |
| Electronegativity | 1.2 – 3.2 |  | 10 |
| 1st ionization energy | 6.5 – 15 | eV | 10 |
| Electron affinity | -0.8 – 2.4 | eV | 10 |
| Density | 0 – 23 | g/$\mathrm{cm}^{3}$ | 10 |
| Weight | 1 – 240 | g/mol | 10 |
| Radius | 0 – 1.65 | $Å$ | 10 |
| Atomic volume | 6.5 – 18 | $\mathrm{cm}^{3}/mol$ | 10 |
| Melting point | -260 – 3500 | $℃$ | 10 |
| Boiling point | -274 – 5600 | $℃$ | 10 |
| $Z_{\mathrm{eff}}$ | 1 – 5.3 |  | 10 |
| Heat of vaporization | 100 – 750 | kJ/mol | 10 |
| Heat of fusion | 6 – 30 | kJ/mol | 10 |
| Polarizability | 25 – 60 | Atomic unit | 10 |
| Resistivity | 0 – 160 | 10^-8^ Ω·m | 10 |
| Atomization energy | 250 – 750 | kJ/mol | 10 |
| Heat capacity | 0.12 – 0.57 | J/g·K | 10 |
| Number of valence electrons | 0 – 10 |  | 10 |
| Number of d electrons | 0 – 10 |  | 10 |


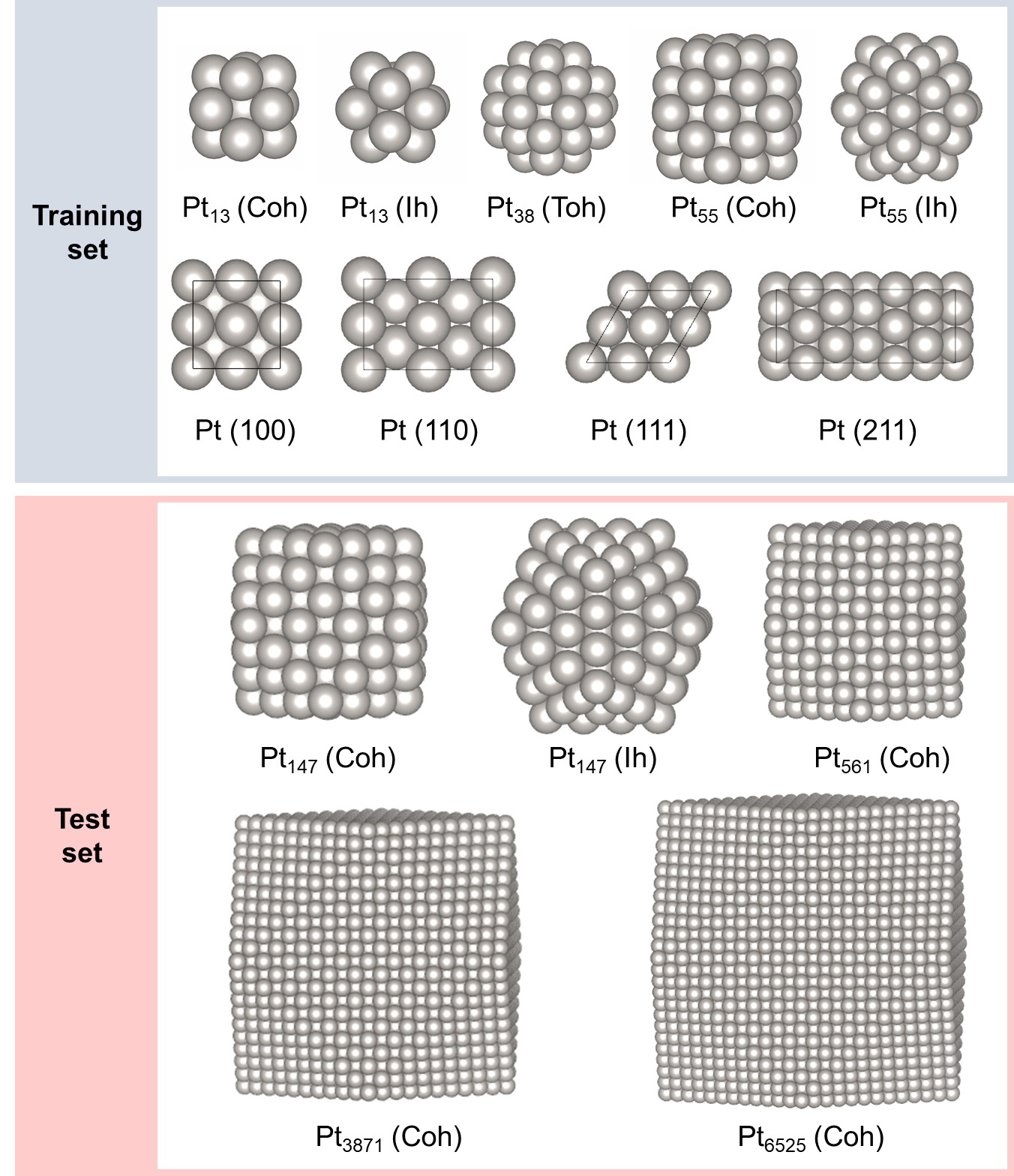


Supplementary Figure S1. NPs used in constructing surface Pourbaix diagram. The NPs in training set is only used for training of BE-CGCNN model. Coh, Ih, and Toh denotes cuboctahedron, icosahedron, and truncated octahedron, respectively.

**Supplementary Table S2. Number of adsorption energy data (M) for each n (n as in NP-(A_ads_)_n_) and each adsorbate (O or OH).**

| **NP** | **Number of adsorption energy data** | |
| --- | --- | --- |
|  | **O** | **OH** |
| Pt_13_ (Ih) | 48 | 49 |
| Pt_13_ (Coh) | 51 | 53 |
| Pt_38_ (Toh) | 47 | 38 |
| Pt_55_ (Ih) | 104 | 109 |
| Pt_55_ (Coh) | 105 | 110 |
| Pt slab | 14 | 8 |
| **Total** | **369** | **367** |

**Supplementary Table S3. Number of O and OH adsorbates in 1ML coverage**

| **NP** | **Number of adsorbates in 1ML coverage** | |
| --- | --- | --- |
|  | **O** | **OH** |
| Pt_13_ (Ih) | 24 | 12 |
| Pt_13_ (Coh) | 24 | 12 |
| Pt_38_ (Toh) | 44 | 32 |
| Pt_55_ (Ih) | 60 | 42 |
| Pt_55_ (Coh) | 72 | 42 |
| Pt_147_ (Ih) | 150 | 92 |
| Pt_147_ (Coh) | 168 | 92 |
| Pt_561_ (Coh) | 440 | 252 |
| Pt_3871_ (Coh) | 1680 | 1002 |
| Pt_6525_ (Coh) | 2400 | 1442 |


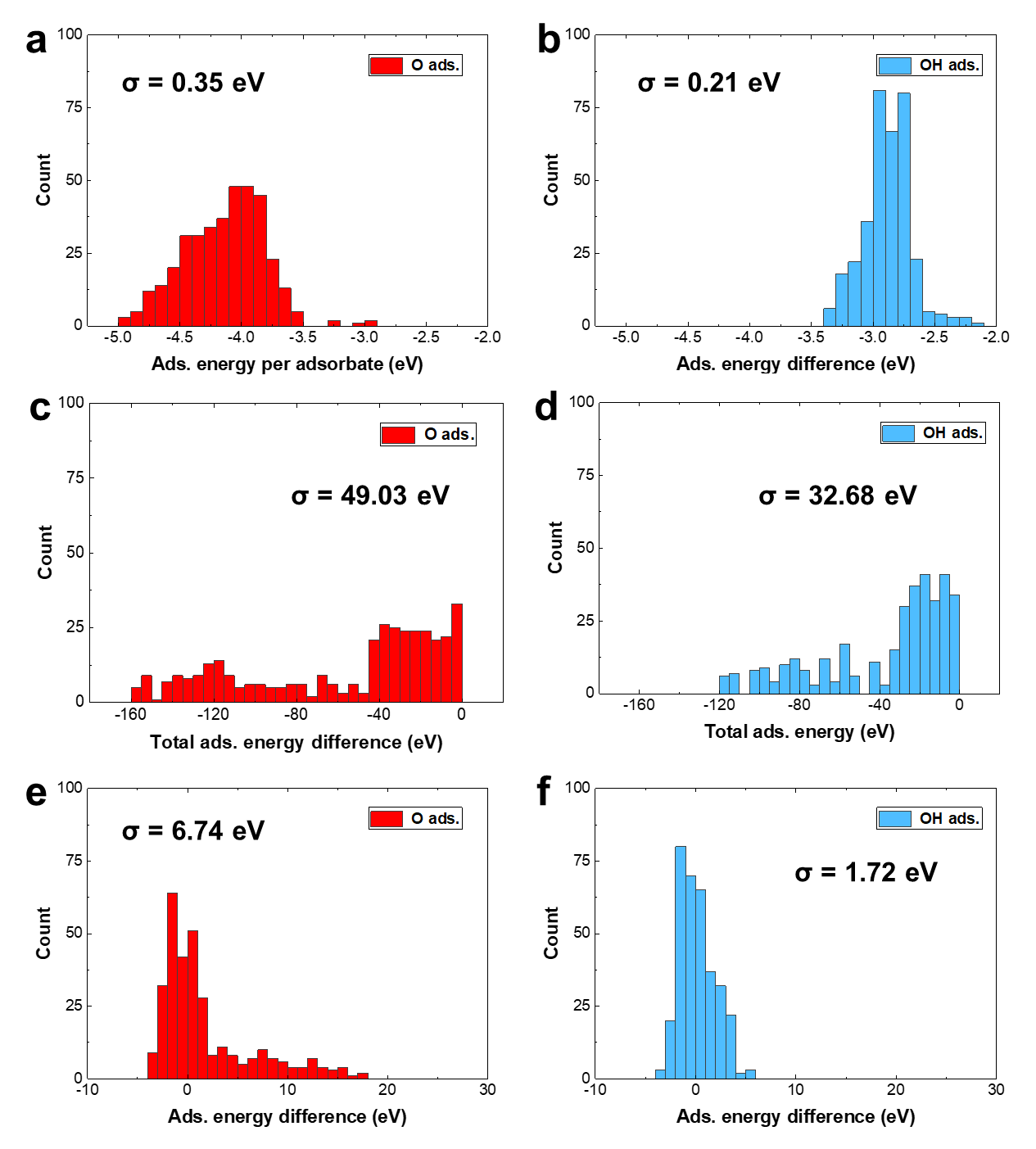


Supplementary Figure S2 The data histogram of different adsorption energy metrics. a, b adsorption energy per adsorbate. c, d total adsorption energy. e, f adsorption energy difference. σ represents the standard deviation of each adsorption energy metrics.


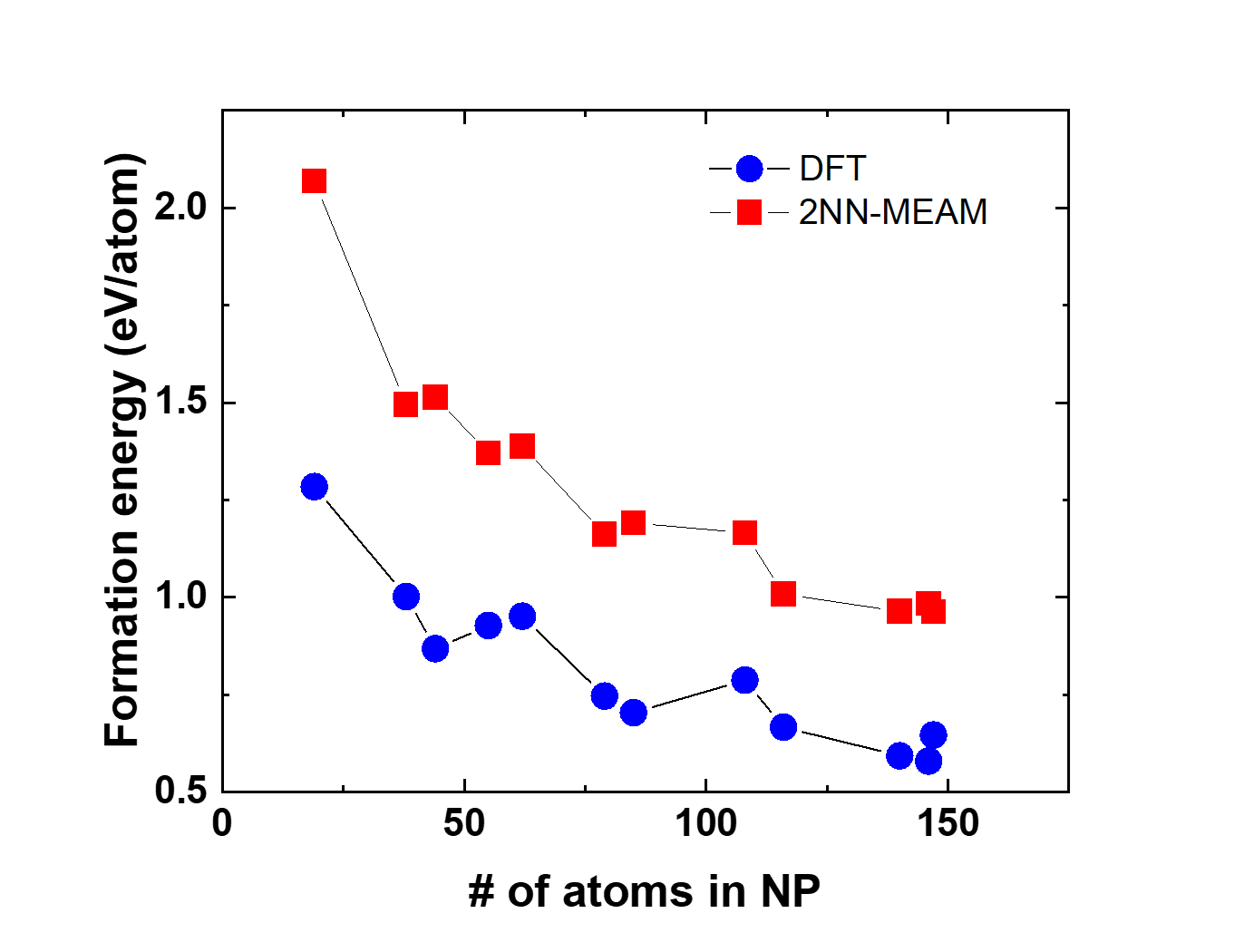


Supplementary Figure S3 Comparison of formation energy of Pt NPs. The atomic energy of Pt bulk was used as a reference to calculate formation energy.


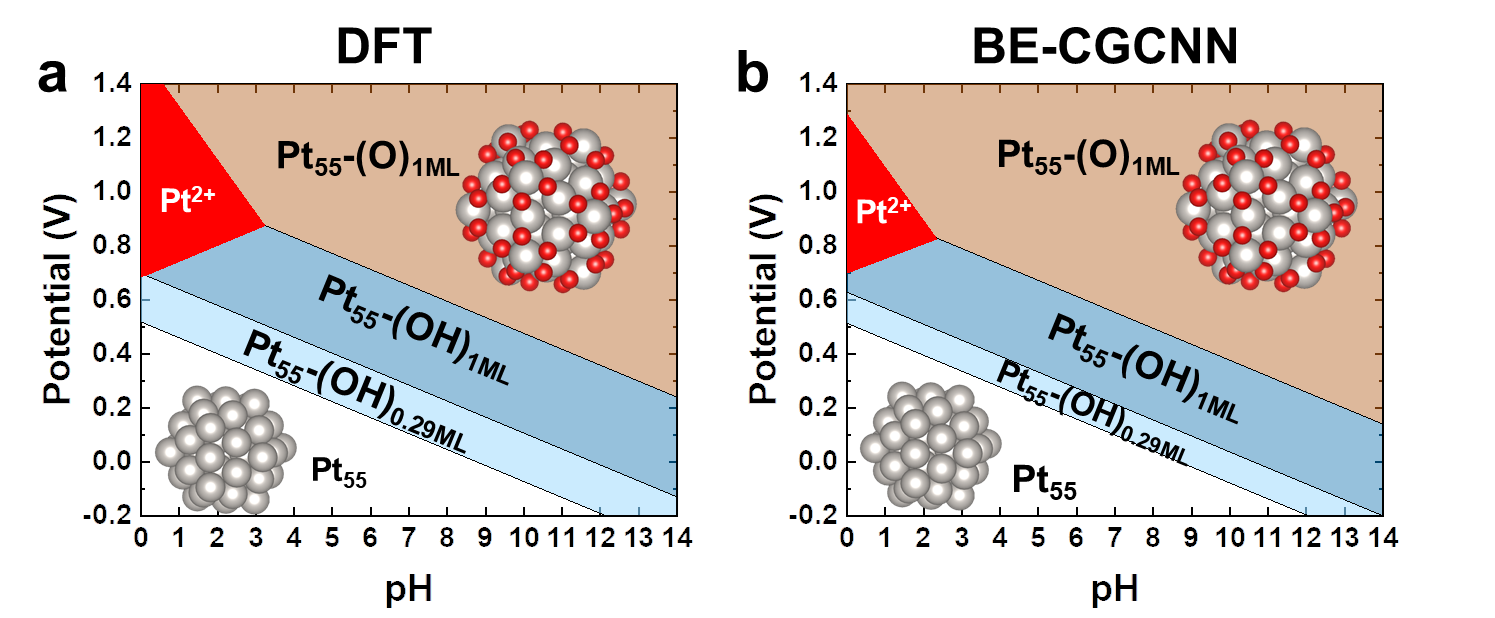


Supplementary Figure S4 Surface Pourbaix diagram of Pt_55_ (Ih) NPs. a Pt_55_ (Ih) constructed by DFT calculation. b Pt_55_ (Ih) consted by ML prediction trained with data of Pt slab, Pt_13_, and Pt_38_. The white, blue, orange, and red shaded area represent bare Pt NPs, OH-covered, O-covered, and Pt dissolution phases, respectively. As the color became darker, more adsorbates are adsorbed.


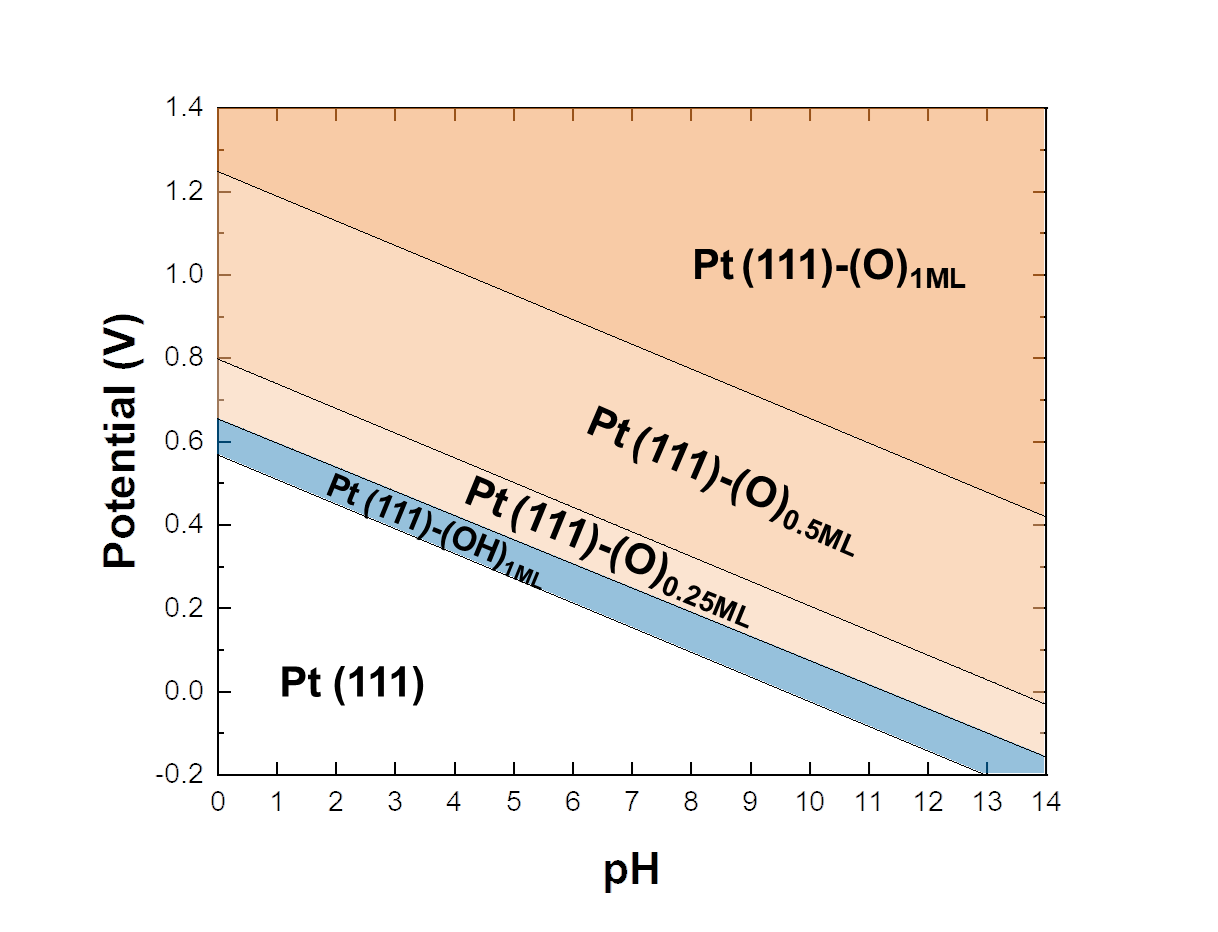


Supplementary Figure S5 Pourbaix diagram of Pt(111) slab constructed by DFT calculation. For the slab calculation, 1×1, √3×√3, and 2×2 surface unit cell was considered, and adsorption on 1×1 surface unit cell was considered as 1ML. The white, blue, and orange shaded area represent bare Pt slab, OH-covered, and O-covered phases, respectively. As the color became darker, more adsorbates are adsorbed.


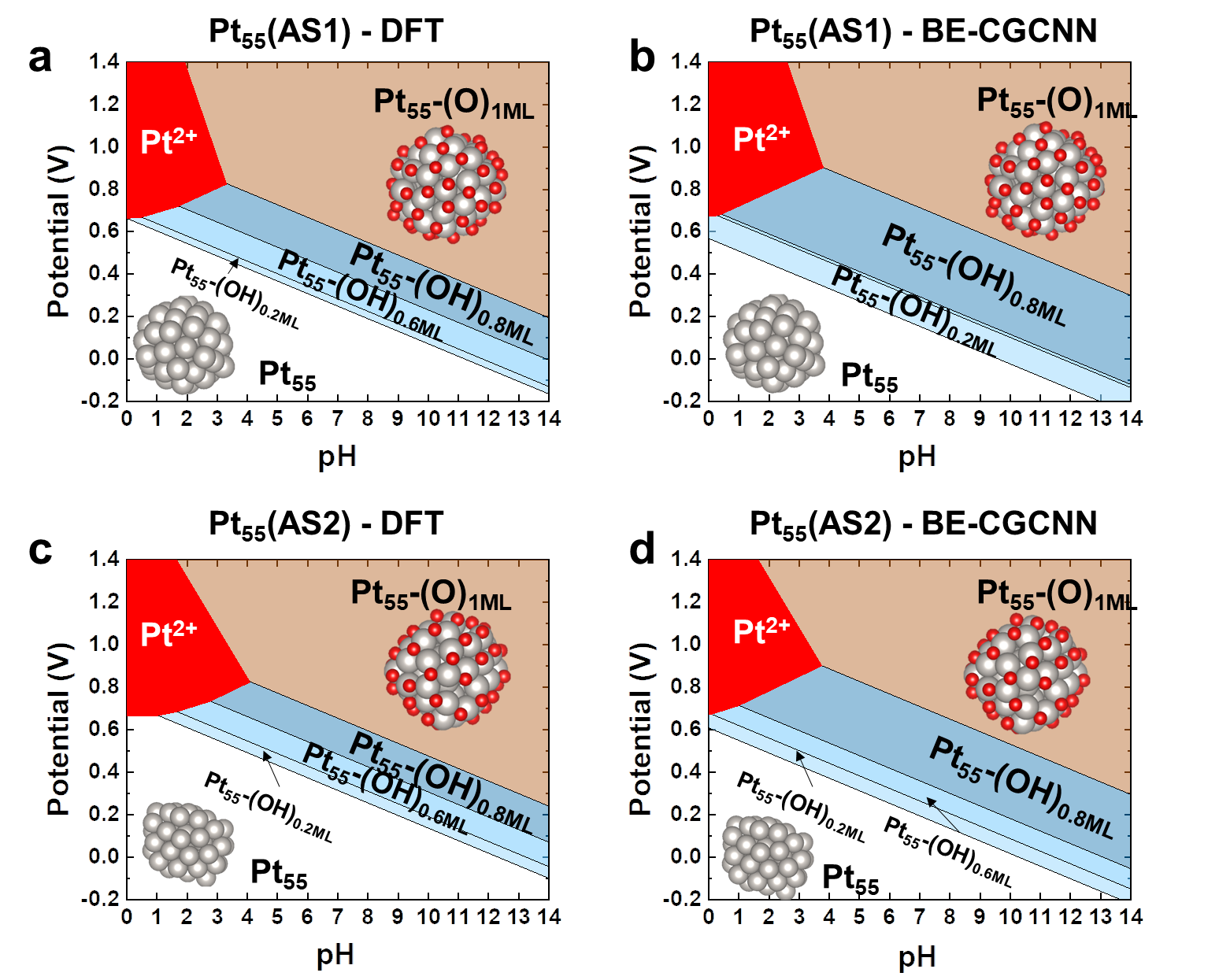


Supplementary Figure S6 Surface Pourbaix diagrams of asymmetric NPs (named as AS1 and AS2) based on density functional theory (DFT) calculation or BE-CGCNN prediction. a Pt_55_ (AS1) based on DFT. b Pt_55_ (AS1) based on BE-CGCNN. c Pt_55_ (AS2) based on DFT. d Pt_55_ (AS2) based on BE-CGCNN. The white, blue, orange, and red shaded area represent bare Pt NPs, OH-covered, O-covered, and Pt dissolution phases, respectively. As the color became darker, more adsorbates are adsorbed. These asymmetric nanoparticles were generated using the heating-and-quenching approach in molecular dynamics simulations (LAMMPS program). The procedure is composed of four steps: (1) prepare a NP cluster composed of 55 atoms, (2) For 200 ps, heat the box up to 1000 K which is higher than the melting temperature of the Pt cluster, (3) For 0.1 ps, rapidly cool down the box to 10 K to main the asymmetric shapes, and (4) finally perform DFT relaxations to obtain local minimum structures. Note that the MD time-step was chosen as 1 fs, and the canonical (NVT) ensemble was used. The box size for the MD simulation was 35×35×35 Å^3^, in which one NP structure was included.


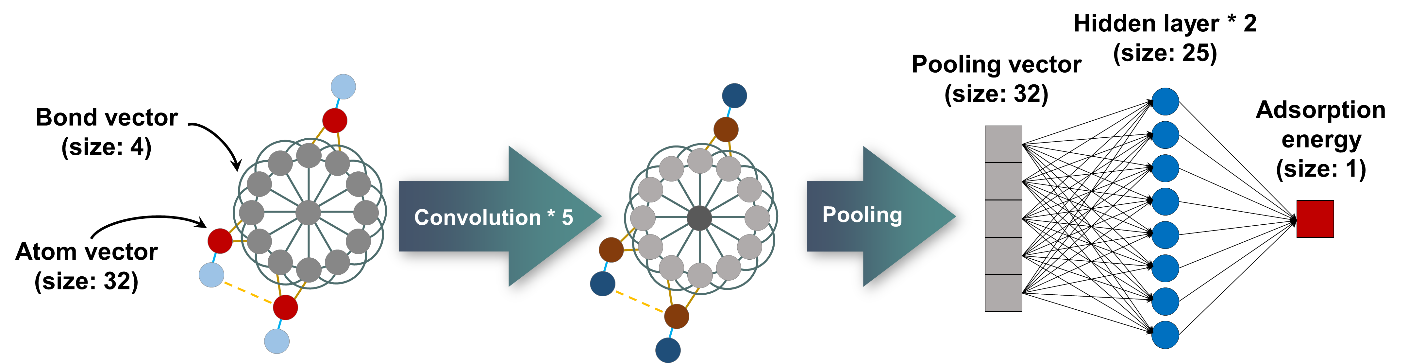


Supplementary Figure S7 Detailed schematic diagram of BE-CGCNN model. Exact number of convolution layer, vector size, and number of node are denoted.


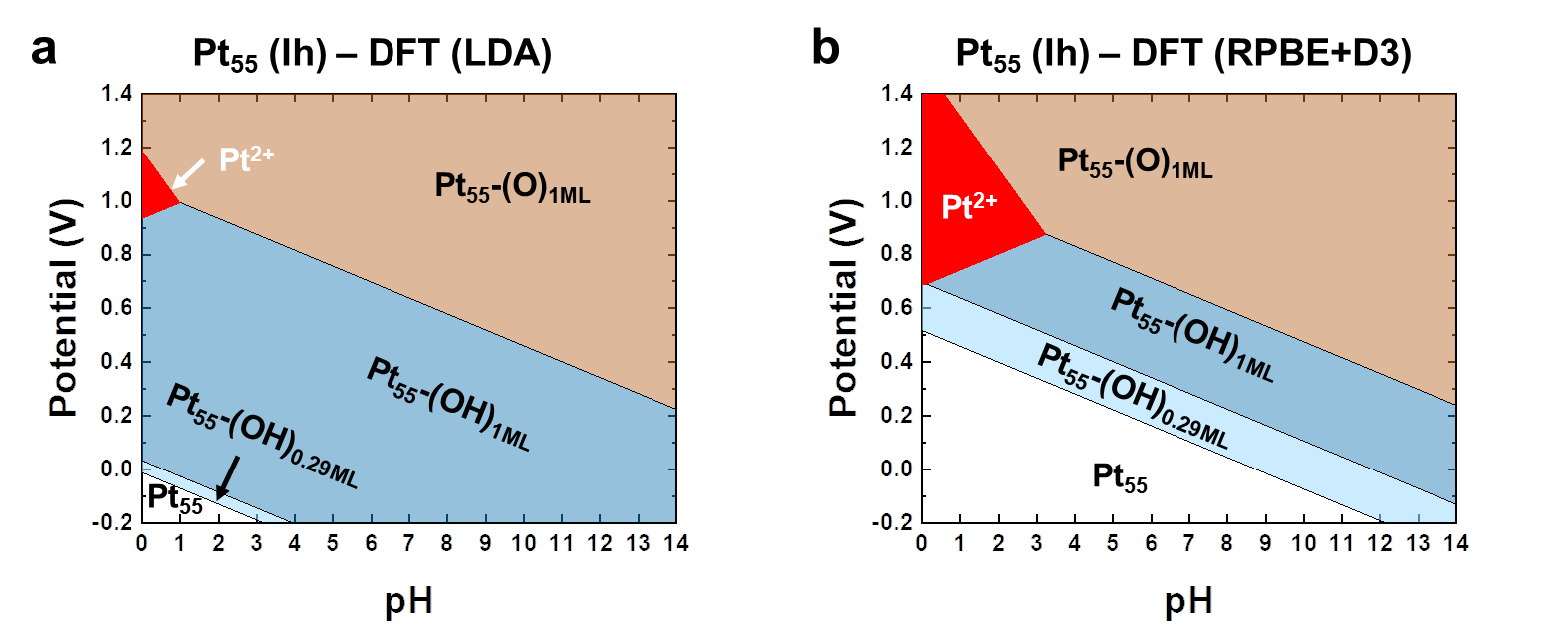


Supplementary Figure S8 Comparison of Pourbaix diagrams of Pt_55_(Ih) obtained by different functionals. a Constructed using LDA functional. b Constructed using RPBE+D3 functional. The white, blue, orange, and red shaded area represent bare Pt NPs, OH-covered, O-covered, and Pt dissolution phases, respectively. As the color became darker, more adsorbates are adsorbed.


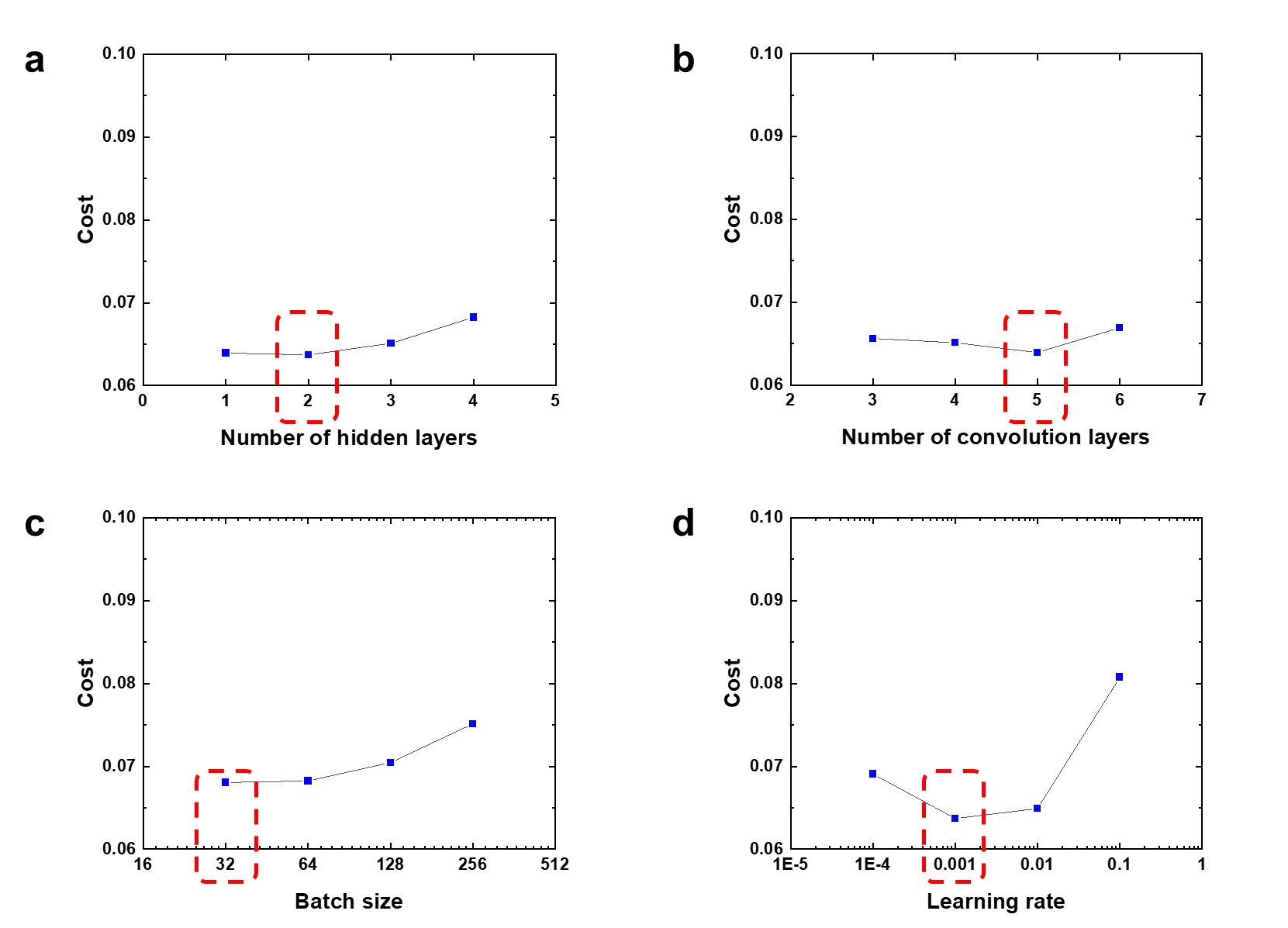


Supplementary Figure S9 Result of hyperparameter fitting. a number of hidden layers. b number of convolution layers. c batch size. d learning rate. Red box indicates the selected parameter values.


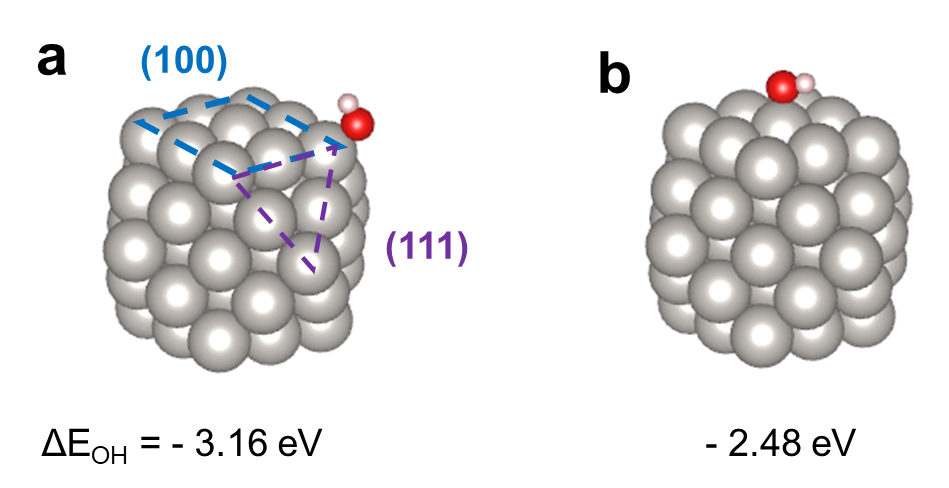


Supplementary Figure S10 OH adsorbed structure of Pt_55_ (Coh). a OH adsorbed on vertex site. b OH adsorbed on terrace site. ΔE_OH_ is adsorption energy of OH, which is calculated as follows: ΔE_OH_=E(Pt_55_-OH) – E(Pt_55_) – E(OH), where E(Pt_55_-OH), E(Pt_55_), and E(OH) are the energies of OH-adsorbed Pt_55_(Coh), bare Pt_55_(Coh), and OH radical, respectively.


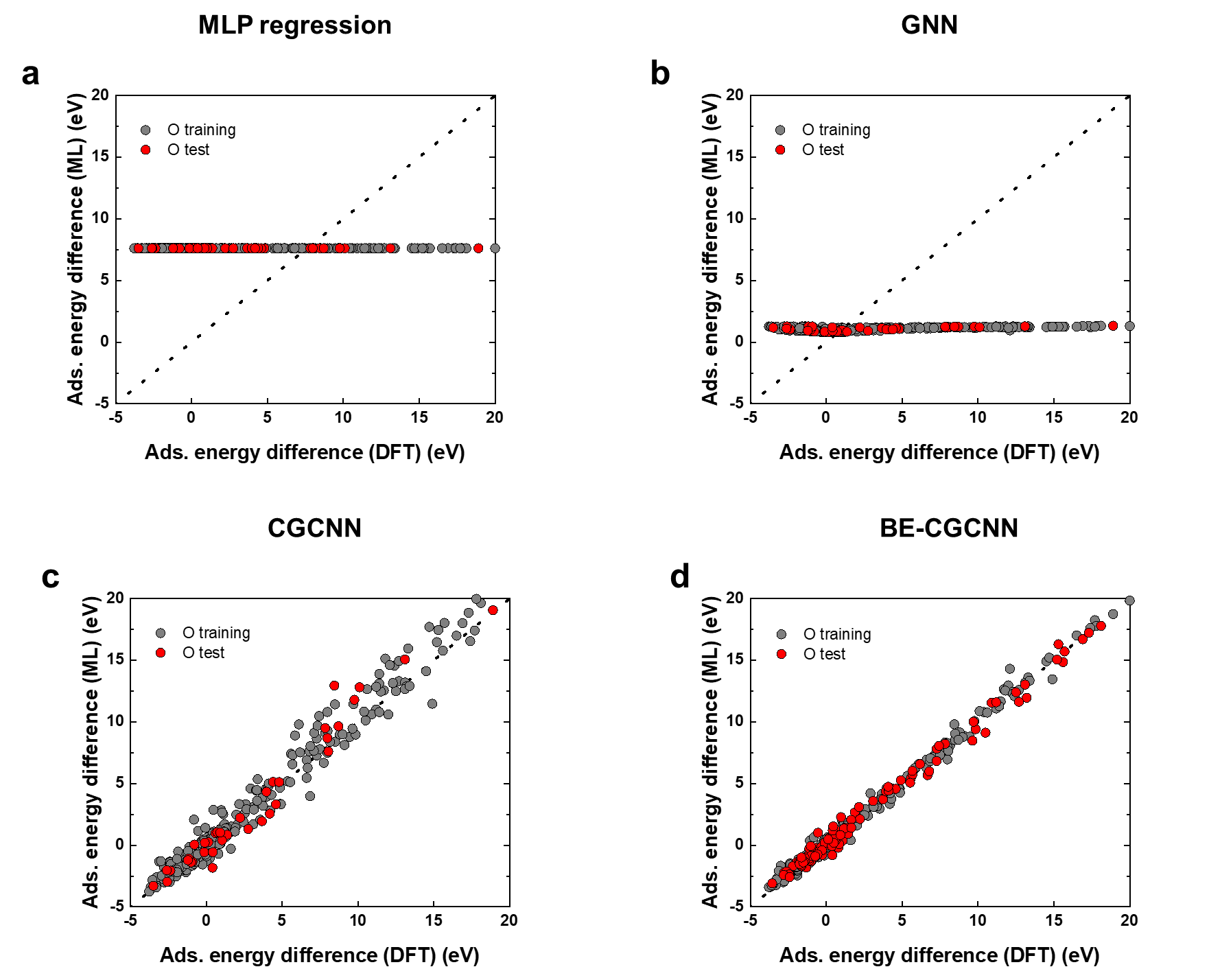


Supplementary Figure S11 Comparative study different models on O adsorption energy difference prediction. a Multi-Layer perceptron (MLP) regression. b Graph neural network (GNN). c CGCNN. d BE-CGCNN models For MLP regression, type of NPs and coverage of adsorbates are used as input features. GNN was produced by replacing convolution layer of CGCNN model. CGCNN is corresponding to ‘without bond type embedding’ model in Table 1.


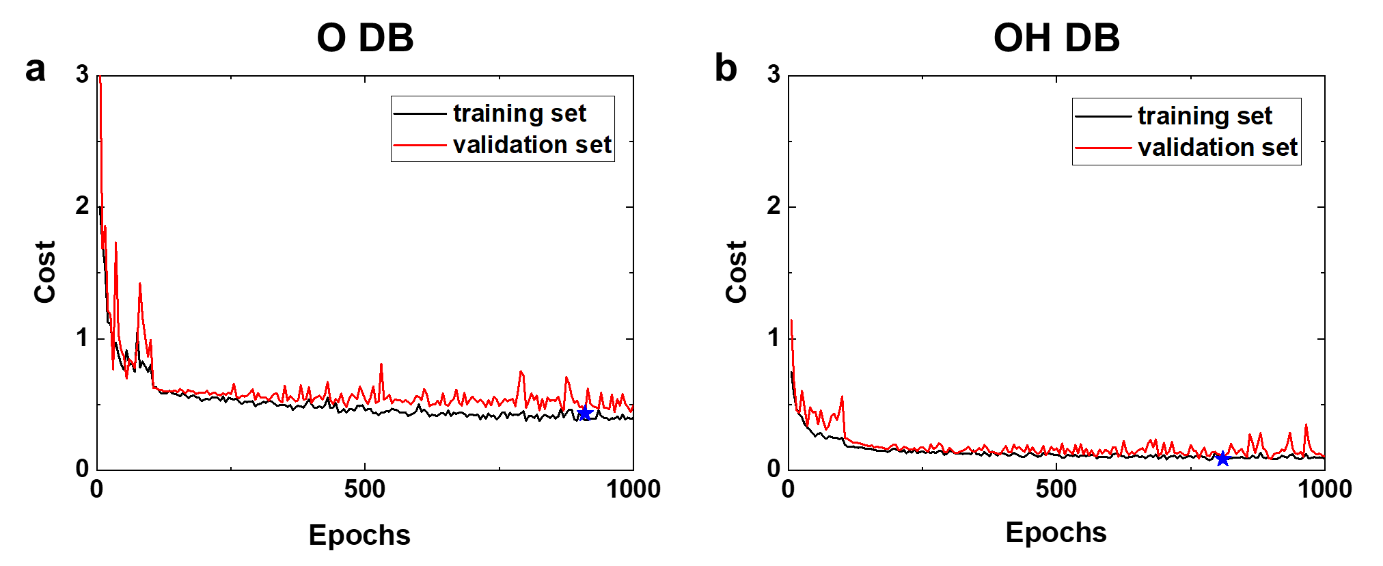


Supplementary Figure S12 Cost of training and validation set at every 5 steps during training process. a Training on O adsorption DB. b Training on OH adsorption DB. The minimum value of the cost of validation set is denoted as a blue star.
